# CircPIP5K1A facilitates gastric cancer progression via miR-376c-3p/ZNF146 axis

**DOI:** 10.1186/s12935-020-1122-5

**Published:** 2020-03-14

**Authors:** Yan Ma, Xiliang Cong, Yiyun Zhang, Xin Yin, Ziyu Zhu, Yingwei Xue

**Affiliations:** 1 Department of Gastroenterological Surgery, Harbin Medical University Cancer Hospital, 150 Haping Road, Harbin, 150081 China; 2 Department of Endoscopy, Harbin Medical University Cancer Hospital, 150 HaPing Road, Harbin, 150081 China

**Keywords:** Gastric cancer, CircPIP5K1A, miR-376c-3p, ZNF146

## Abstract

**Background:**

Recently, many emerging circular RNAs (circRNAs) have been studied in human malignancies, including gastric cancer (GC). Researches concerning cancers have revealed that aberrant expression of circRNAs play a big part in tumorigenesis and development of diverse malignant tumors. Although hsa_circ_0014130 (circPIP5K1A) has been confirmed to be closely related to non-small cell lung cancer (NSCLC) progression, the knowledge of its function on GC progression remains unclear. Therefore, it is of great interest to uncover the underlying role of circPIP5K1A in GC.

**Methods:**

The expression and characteristic of circPIP5K1A were separately analyzed by RT-qPCR, nucleic acid electrophoresis, RNase R and Actinomycin D treatment. CCK-8, colony formation, EdU, transwell, TUNEL, flow cytometry, luciferase reporter, RIP and RNA pull-down assays were employed to testify the regulatory role of circPIP5K1A in GC.

**Results:**

In current study, circPIP5K1A, featured with closed-loop structure, was proved to be highly expressed in tissues and cells of GC. Loss-of-function assays depicted that silencing circPIP5K1A suppressed GC development. Follow-up mechanism tests unveiled that circPIP5K1A bound with miR-376c-3p and inhibition of miR-376c-3p reversed circPIP5K1A downregulation-mediated effect on GC progression. Additionally, ZNF146 was verified to be the downstream molecule of circPIP5K1A/miR-376c-3p axis in modulating GC progression.

**Conclusions:**

circPIP5K1A stimulates GC progression by sponging miR-376c-3p to upregulate ZNF146 expression.

## Background

Gastric cancer (GC), one of the most prevalent fatal neoplasms, ranks the second factor that leads to the occurrence of cancer-related death in human all over the world [1–3]. GC has been frequently diagnosed with high incidence in many Asian countries, especially China and Japan [4, 5]. In the past years, with rapid progress in diagnostic apparatus and therapeutic methods, the morbidity and mortality ratio of GC appeared to be on a stably downward trend [6]. However, because of the frequent recurrence and metastasis of malignancy, the prognosis of GC patients at advanced stage is still poor, characterized by a low 5-year overall survival rate [7]. The tumorigenesis and development of GC are extremely complicated biological courses regulated by aberrant expression of oncogenes or dysregulation of anti-tumor genes [8–11]. Thus far, the knowledge of potential molecular mechanisms in GC remains poorly understood. To identify effective targeted therapies, further exploration and elucidation on the underlying molecular mechanisms involved in GC are urgent events for investigators.

Circular RNAs (circRNAs), a newly identified type of endogenous non-coding RNAs characterized with closed-loop structure without 5′ to 3′ polarity, are generated via end-to-end combining of RNA transcription fragments [12–14]. Circular transcripts were once regarded as abnormal RNA splicing or particular pathogens despite the fact that they have been known for more than 40 years [15]. Besides, circRNAs, different from other non-coding RNAs, are very stable because of their loop structure [16]. Multiple studies have suggested that circRNAs are related to a variety of biological processes of diverse tumors, including GC. For instance, circRNA hsa_circ_0052112 accelerates breast cancer progression by sponging miR-125a-5p [17]. CircRNA hsa_circ_0000064 exerts critical role in lung cancer cell proliferation and metastasis [18]. CircRNA circ-LDLRAD3 is associated with pancreatic cancer tumorigenesis [19]. CircRNA-ZFR regulates GC progression via miR-130a/107/PTEN axis [20]. CircPIP5K1A is an identified circRNA and its critical role in non-small cell lung cancer (NSCLC) has been mentioned in a previous research [21]. However, the underlying regulatory mechanism of circPIP5K1A in GC needs further elucidation.

This current study was designed to explore the specific regulatory mechanism of circPIP5K1A in GC. And the results elucidated that circPIP5K1A facilitates GC progression via miR-376c-3p/ZNF146 axis, hinting that circPIP5K1A can be applied as a new and efficient biomarker in researches concerning GC treatment.

## Methods

### Human tissue samples

49 pairs of cancerous and paired non-cancerous tissues were collected from GC patients who underwent surgical resections in Harbin Medical University Cancer Hospital from Jan 2012 and Dec 2017. All patients did not receive any treatment before operation. All fresh samples were stored in liquid nitrogen at − 80 °C. Patients signed the relevant informed consent and the study was allowed by the Ethics Committee of Harbin Medical University Cancer Hospital.

### Cell culture

GC cells (BGC-823, MGC-803, HGC-27, MKN-45) and normal human gastric epithelial cell line (GES-1) were gained from Chinese Academy of Sciences (Shanghai, China). Cells were cultivated in RPMI-1640 media (Gibco, Carlsbad, USA) supplied with 10% FBS (Gibco) in incubator including 5% CO_2_ at 37 °C.

### Cell transfection

Cells were placed into 6-well plates and developed to 70% confluence. For the knockdown of circPIP5K1A, specific sh-circPIP5K1A#1/2 and corresponding control sh-NC were constructed by Gene Pharma (Shanghai, China). The pcDNA3.1/ZNF146, pcDNA3.1/circPIP5K1A and the empty pcDNA3.1 (+) circRNA Mini Vector were obtained from Gene Pharma. Meanwhile, specific miR-376c-3p inhibitor and NC inhibitor, miR-376c-3p mimics and NC mimics were also synthesized by Gene Pharma. All plasmids were transfected into cells by using Lipofectamine 2000 kit (Invitrogen, NY, USA).

### Nucleic acid electrophoresis

Utilizing agarose gels together with TE buffer (Thermo Fisher Scientific, Waltham, MA, USA), the cDNA and gDNA PCR products of circPIP5K1A were analyzed. Afterwards, DL600 (KeyGen, Nanjing) was used as the DNA marker. The electrophoretic voltage was 110 volts. DNA was separated for 30 min. UV irradiation was adopted to test the results.

### Quantitative real-time PCR (RT-qPCR)

The experiment was applied to monitor the relative expression of RNA in samples from cells or tissues [22].

### Actinomycin D (ActD) and RNase R treatment assays

For RNase R assay, total RNA was cultivated for 20 min at 37 °C with 3U/ug of RNase R (Epicentre Biotechnologies, WI, USA). For ActD assay, ActD was bought from Sigma-Aldrich (Milan, Italy) and extracted RNA was treated with ActD (5 mg/ml) in indicated time points and then RT-qPCR analyses were performed. The two assays were conducted in triplicate.

### Cell viability assay

MKN-45 cells were seeded into 96-well plates and the CCK-8 reagent was added to every well at specific time points. Following cultivation for 4 h, OD was analyzed by a spectrometer reader (Olympus, Tokyo, Japan).

### Colony formation assay

After transfection, GC cells were maintained in 6-well plates and incubated for 2 weeks. Later, cells in each well were fixed and dyed by methanol (Solarbio, Beijing, China) or crystal violet (Solarbio) for 10 min or 5 min, respectively.

### Luciferase reporter assay

CircPIP5K1A fragment containing putative miR-376c-3p binding site was sub-cloned into pmirGLO vector (Promega, Carlsbad, USA) and named circPIP5K1A-WT. Then we made a circPIP5K1A-Mut which the putative miR-376c-3p binding site was mutated. The 2 reporter plasmids were co-transfected with miR-376c-3p mimics or NC mimics into MKN-45 or HGC-27 cells, separately. The activities of luciferase were measured by a dual-luciferase reporter system (promega) at 48 h after transfection.

### In vivo experiment

MKN-45 cells were injected into every nude mouse. Finally, the mice were killed and pictures of their tumors were collected. The tumor volume and weight were calculated. These experiments gained permission from the Committee on the Ethics of Animal Experiments of Harbin Medical University Cancer Hospital.

### Transwell assay

Transwell chamber which was coated with or without Matrigel (BD Biosciences, Massachusetts, USA) was applied to measure cell invasion or migration, respectively. MKN-45 or HGC-27 cells in serum-free media were placed into the top chamber. Then, media with 10% FBS was added into the basolateral chamber. 48 h later, invaded or migrated cells were fixed in methanol (Solarbio), stained with 0.1% crystal violet (Solarbio) and counted via a microscope (Olympus).

### TUNEL assay

The apoptosis of HGC-27 or MKN-45 cells were assessed by the use of TUNEL Apoptosis Kit (Invitrogen) in line with manufacturer’s recommendations. The cells mentioned above were stained by DAPI (Thermo Fisher Scientific, Waltham, MA, USA) or Merge (Thermo Fisher Scientific). Then, cells were surveyed and captured by fluorescence microscopy (Olympus).

### Cell apoptosis assay

The experimental procedure was progressed as previously depicted and the details were as follows [23].

### RNA pull down assay

In short terms, biotin probe and non-biotin probe of circPIP5K1A were synthesized by GenePharma and then treated with M-280 Streptavidin magnetic beads (Invitrogen) to construct probe-coated beads. Then, cells of MKN-45 or HGC-27 were incubated with probe-coated beads overnight at 4 °C. RNA complexes adhered the beads after cleaning were evaluated by RT-qPCR.

### Immunohistochemical (IHC) staining

The steps of this assay was performed the same as previous described [24].

### EdU incorporation assay

Cells were plated into 96-well plates and (100 μL) EdU reagent was added to every well to be cultured for 2 h. After cleaning with PBS (Solarbio), cells were dyed with 100 μL of Apollo in the dark for 30 min. Then cells were fixed, decolored and permeated after it. Subsequently, the cells were cultivated with 100 μL of 1 × DAPI reaction solution followed by viability determination using a fluorescent microscope (Olympus).

### RIP assay

This assay was conducted by using a Thermo Fisher RIP kit (Thermo Fisher Scientific) following the supplier’s instructions. At a word, cells were lysed in RIP lysis buffer, and RNAs magnetic beads were conjugated with a human anti-Ago2 antibody or with anti-IgG. Subsequently, the retrieved RNA was evaluated by RT-qPCR.

### Subcellular fractionation

This experiment was conducted using the PARIS Kit (Life Technologies, Beijing, China). Based on the manufacturer’s protocols, the cells were separated into nuclear and cytoplasmic fractions before undergoing the RNA isolation procedure. Besides, GAPDH or U6 was seen as cytoplasmic and nuclear control, separately.

### Statistical analysis

Data were denoted as mean ± SD. Statistical analysis was conducted using the SPSS (Chicago, USA) and GraphPad Prism 5 software (San Diego, CA). Significance of the variance between two or several groups was evaluated by Student’s t test or ANOVA. P < 0.05 has statistically significance. The experiments were conducted thrice.

## Results

### CircPIP5K1A is markedly elevated and characterized with loop structure

**Fig. 1:**
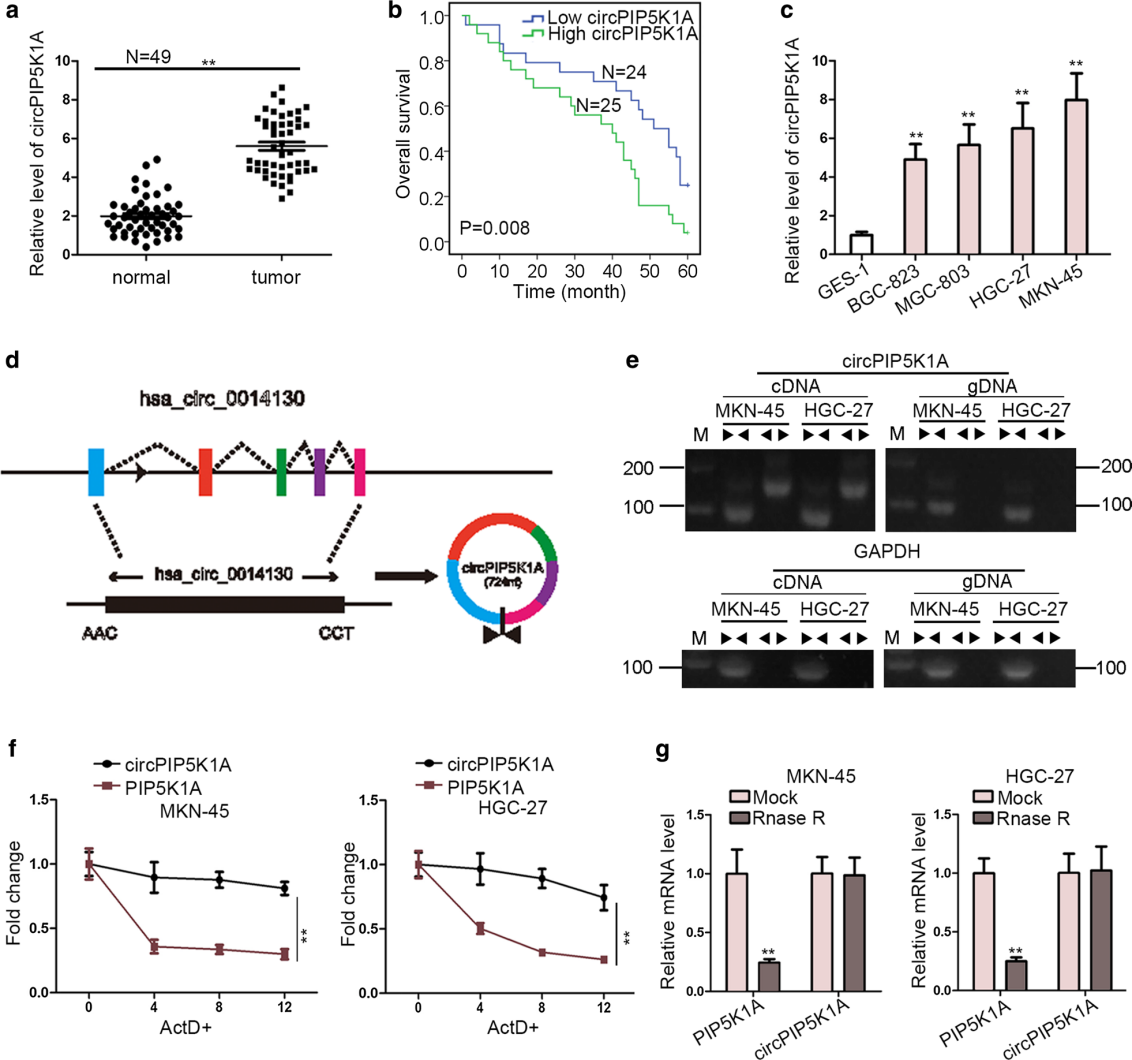
CircPIP5K1A is markedly elevated and characterized with loop structure in GC. **a** The expression of circPIP5K1A in 49 pairs GC tissues and contiguous normal tissues was detected by RT-qPCR. **b** Overall survival curve of GC patients with high or low circPIP5K1A expression, determined by Kaplan–Meier analysis and log-rank test. **c** The expression of circPIP5K1A in GC cell lines (BGC-823, MGC-803, HGC-27 and MKN-45) and normal GES-1 cells was examined by RT-qPCR. **d** The genomic location and splicing pattern of circPIP5K1A was displayed. **e** Nucleic acid electrophoresis delineated that divergent primers amplified circPIP5K1A from cDNA, but not from gDNA. GAPDH was used as a negative control. **f** The resistance of circPIP5K1A and PIP5K1A mRNA to ActD was detected by RT-qPCR assay in MKN-45 and HGC-27 cells. **g** RT-qPCR assay was carried out to determine the abundance of circPIP5K1A and linear PIP5K1A mRNA in MKN-45 and HGC-27 cells treated with RNase R (normalized to mock treatment). ^**^P < 0.01

Notwithstanding the confirmed involvement of circPIP5K1A in NSCLC progression [21], the potential role of it in GC still needs exploring. To achieve this, the first to do was to understand the expression level of circPIP5K1A in GC tissues and cells. In comparison with contiguous normal tissues, a conspicuous high expression of circPIP5K1A in 49 pairs GC tissues was observed (Fig. 1a). More importantly, we discovered that high circPIP5K1A expression seemed to have strong associations with big tumor diameter, advanced stage and distant metastasis (Table 1). Additionally, it was suggested that poorer prognosis was more likely to be occurred in GC patients with higher circPIP5K1A level (Fig. 1b). Meanwhile, RT-qPCR analysis obtained a dramatic up-regulation of circPIP5K1A in GC cell lines (BGC-823, MGC-803, HGC-27 and MKN-45) compared with GES-1 cells (Fig. 1c). Figure 1d exhibited the genomic location and splicing pattern of circPIP5K1A (hsa_circ_0014130), which was derived from the host gene PIP5K1A. However, whether circPIP5K1A was featured with loop structure required further confirmations. As displayed in Fig. 1e, divergent primers was capable of generating the circular isoform of circPIP5K1A from cDNA rather than from gDNA, while convergent primers was able to amplify the linear isoform of circPIP5K1A from both cDNA and gDNA in MKN-45 and HGC-27 cells. Additionally, after being treated with ActD, PIP5K1A mRNA was more likely to be degraded, whereas circ-PIP5K1A turned out to be more stable and resistant to ActD in MKN-45 and HGC-27 cells (Fig. 1f). Besides, the mRNA expression of PIP5K1A was remarkably cut down whereas the level of circPIP5K1A showed no distinct change in MKN-45 and HGC-27 cells under Rnase R treatment (Fig. 1g).

**Table 1:** Relationship of circPIP5K1A expression in cancer tissues with clinicopathological factors of gastric cancer patients (n = 49)

Variable	CIRCPIP5K1A expression		P value
	Low	High	
Age			0.778
< 60	12	11	
≥ 60	12	14	
Gender			0.567
Male	11	9	
Female	13	16	
Diameter			0.001**
<5	14	3	
≥5	10	22	
Stage			0.001**
Early	16	5	
Advanced	8	20	
Differentiation			0.742
Well/Moderate	5	18	
Poor	19	7	
Distal metastasis			0.010*
M0	16	7	
M1	8	18	

Low/high by the sample median. Pearson χ^2^ test

^*^P < 0.05, ^**^P < 0.01 was considered to be statistically significant

### Decreased expression of circPIP5K1A restrains GC progression

**Fig. 2:**
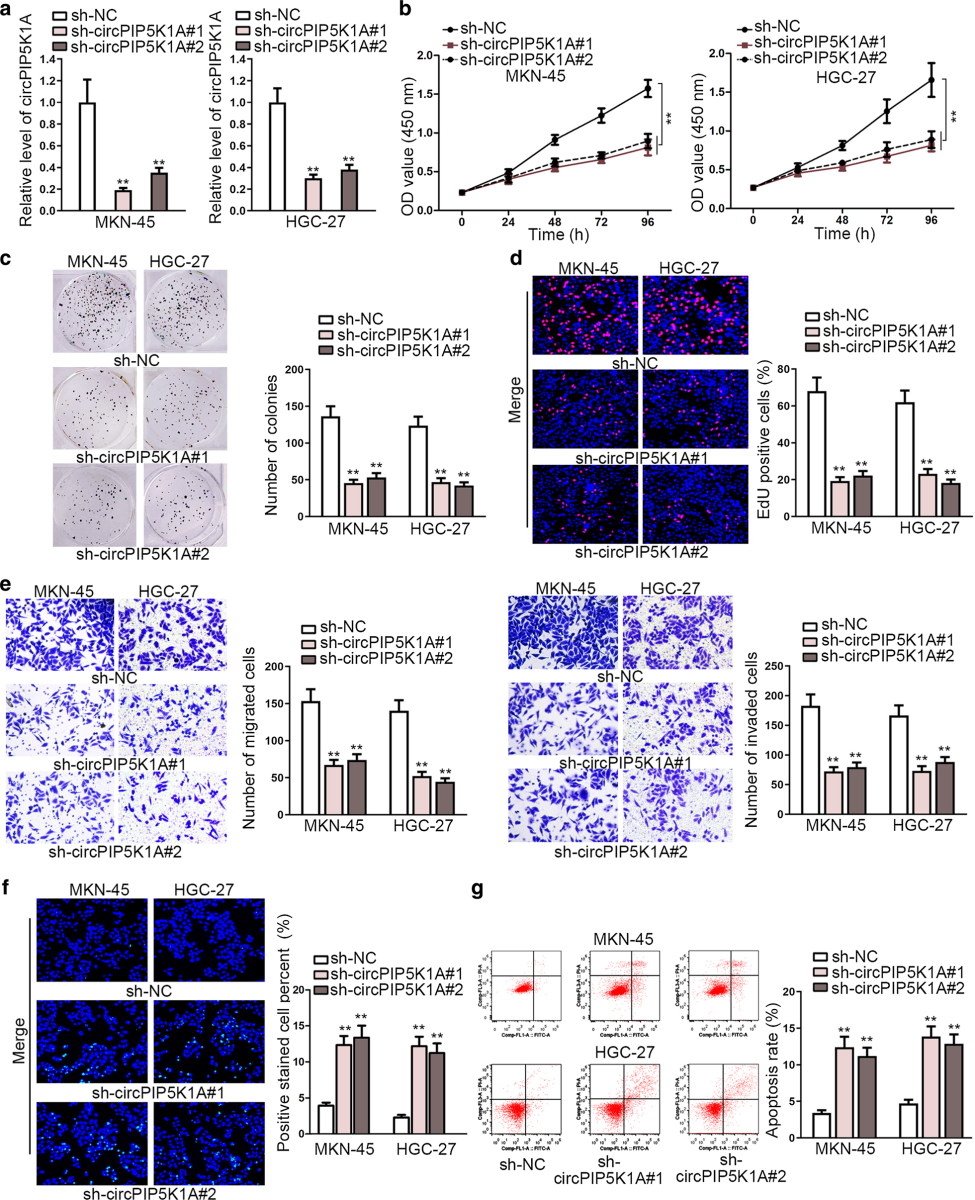
Decreased expression of circPIP5K1A restrains GC progression. **a** RT-qPCR was used to examine the knockdown efficiency of circPIP5K1A in MKN-45 and HGC-27 cells. **b–d** CCK-8, colony formation together with EdU assays were applied to measure cell proliferative ability in MKN-45 and HGC-27 cells transfected with sh-circPIP5K1A#1/2 or sh-NC. **e** The capability of cell migration and invasion was evaluated by transwell assay in transfected cells. **f**–**g** TUNEL and flow cytometry analyses were utilized to assess the apoptosis of transfected cells. ^**^P < 0.01

Subsequently, loss-of-function experiments were exploited to probe into the biological role of circPIP5K1A in GC. Prior to this purpose, RT-qPCR was utilized to examine the knockdown efficiency of shRNAs against circPIP5K1A. As illustrated in Fig. 2a, the expression of circPIP5K1A was significantly downregulated in sh-circPIP5K1A#1/2-transfected cells compared with normal control. Cell proliferation assays delineated an evidently attenuated ability of cell proliferation caused by circPIP5K1A knockdown in MKN-45 and HGC-27 cells (Fig. 2b–d). It was then depicted by transwell assay that in both MKN-45 and HGC-27 cells, circPIP5K1A depletion led to a weakened capability of cell migration and invasion (Fig. 2e). Furthermore, TUNEL and flow cytometry analyses revealed that downregulation of circPIP5K1A induced the apoptosis of MKN-45 and HGC-27 cells (Fig. 2f–g).

### CircPIP5K1A sponges miR-376c-3p in GC

**Fig. 3:**
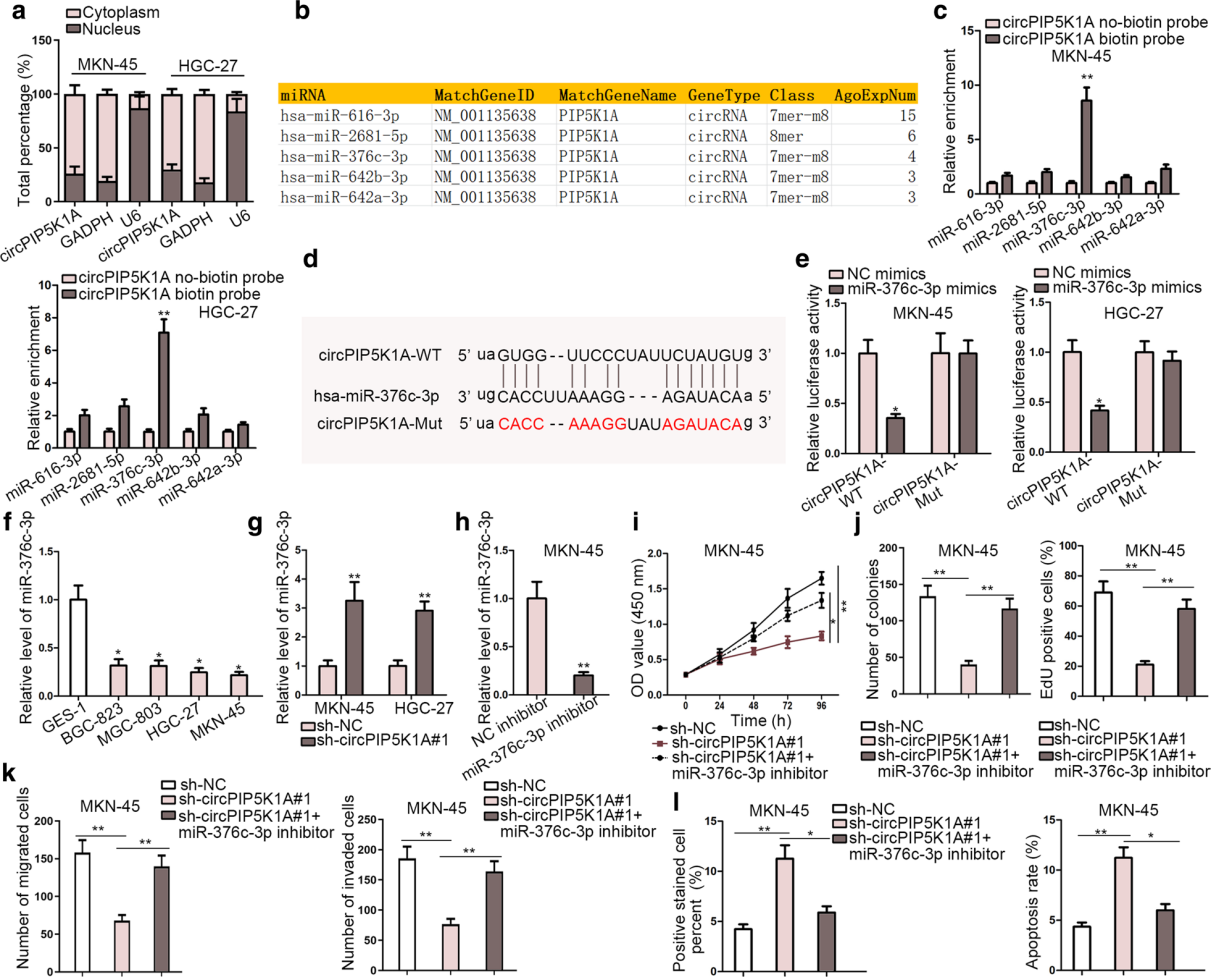
CircPIP5K1A sponges miR-376c-3p in GC. **a** Subcellular fractionation assay was conducted to detect the distribution of circPIP5K1A in MKN-45 and HGC-27 cells. **b** Five miRNAs (miR-616-3p, miR-2681-5p, miR-376c-3p, miR-642b-3p and miR-642a-3p) was obtained from starBase. **c** The binding ability between circPIP5K1A and the above mentioned miRNAs was confirmed by RNA pull-down assay. **d** On the basis of starBase, there existed a binding site between circPIP5K1A and miR-376c-3p. **e** Luciferase reporter assay validated the interaction between circPIP5K1A and miR-376c-3p. **f** The expression of miR-376c-3p in GC cell lines and normal GES-1 cells was examined by RT-qPCR. **g** The expression of miR-376c-3p was detected by RT-qPCR in MKN-45 and HGC-27 cells transfected with sh-circPIP5K1A#1 or sh-NC. **h** The inhibitory efficiency of miR-376c-3p was evaluated by RT-qPCR assay in MKN-45 and HGC-27 cells. **i**–**j** CCK-8, colony formation as well as EdU analyses were utilized to examined the proliferation ability of transfected cells. **k** The capability of cell migration and invasion was evaluated by transwell. **l** The apoptosis ability of transfected cells was analyzed by TUNEL and flow cytometry assays. ^*^P < 0.05, ^**^P < 0.01

To further understand how circPIP5K1A exerted crucial function on the tumorigenesis of GC, subcellular fractionation assay was performed at first to detect the cellular localization of it in GC cells. According to the result, circPIP5K1A appeared to be mainly located in the cytoplasm of GC cells (Fig. 3a). Thereby, we made a bold speculation that circPIP5K1A might post-transcriptionally modulate gene expression by functioning as a competing endogenous RNA (ceRNA) in GC. To testify the speculation, starBase was used for discovery of the candidate miRNAs that were likely to bind with circPIP5K1A and then five miRNAs (miR-616-3p, miR-2681-5p, miR-376c-3p, miR-642b-3p and miR-642a-3p) were screened out, as listed in Fig. 3b. Subsequently, RNA pull-down assay indicated that miR-376c-3p was notably enriched in circPIP5K1A biotin probe group. In regards to other four miRNAs, there exhibited no apparent change in different groups (Fig. 3c). Herein, miR-376c-3p was chosen to be studied in following experiments. On the basis of starBase, there existed a binding site between circPIP5K1A and miR-376c-3p (Fig. 3d). In addition, the luciferase activity of pmirGLO-circPIP5K1A-WT was conspicuously lowered by overexpressing miR-376c-3p while no obvious change of the luciferase activity of pmirGLO-circPIP5K1A-Mut could be perceived in different groups (Fig. 3e). Moreover, compared with normal GES-1 cells, an evident down-regulation of miR-376c-3p in GC cell lines was obtained through RT-qPCR analysis (Fig. 3f). Furthermore, knocking down circPIP5K1A markedly increased the expression of miR-376c-3p in MKN-45 and HGC-27 cells (Fig. 3g). To explore whether miR-376c-3p was the mediator for the function of circPIP5K1A in GC, rescue assays were conducted after confirming the inhibition efficiency of miR-376c-3p inhibitor by RT-qPCR analysis (Fig. 3h). Later on, results of CCK-8, colony formation as well as EdU experiments elucidated that miR-376c-3p inhibition could largely rescue circPIP5K1A silence-mediated suppressive function on cell proliferation (Fig. 3i–j). Similarly, miR-376c-3p suppression could reverse the restraining effect of circPIP5K1A depletion on cell migration and invasion (Fig. 3k). Furtherly, inhibition of miR-376c-3p countervailed the promoting function of circPIP5K1A downregulation in cell apoptosis (Fig. 3l).

### ZNF146 competed with circPIP5K1A for binding with miR-376c-3p

**Fig. 4:**
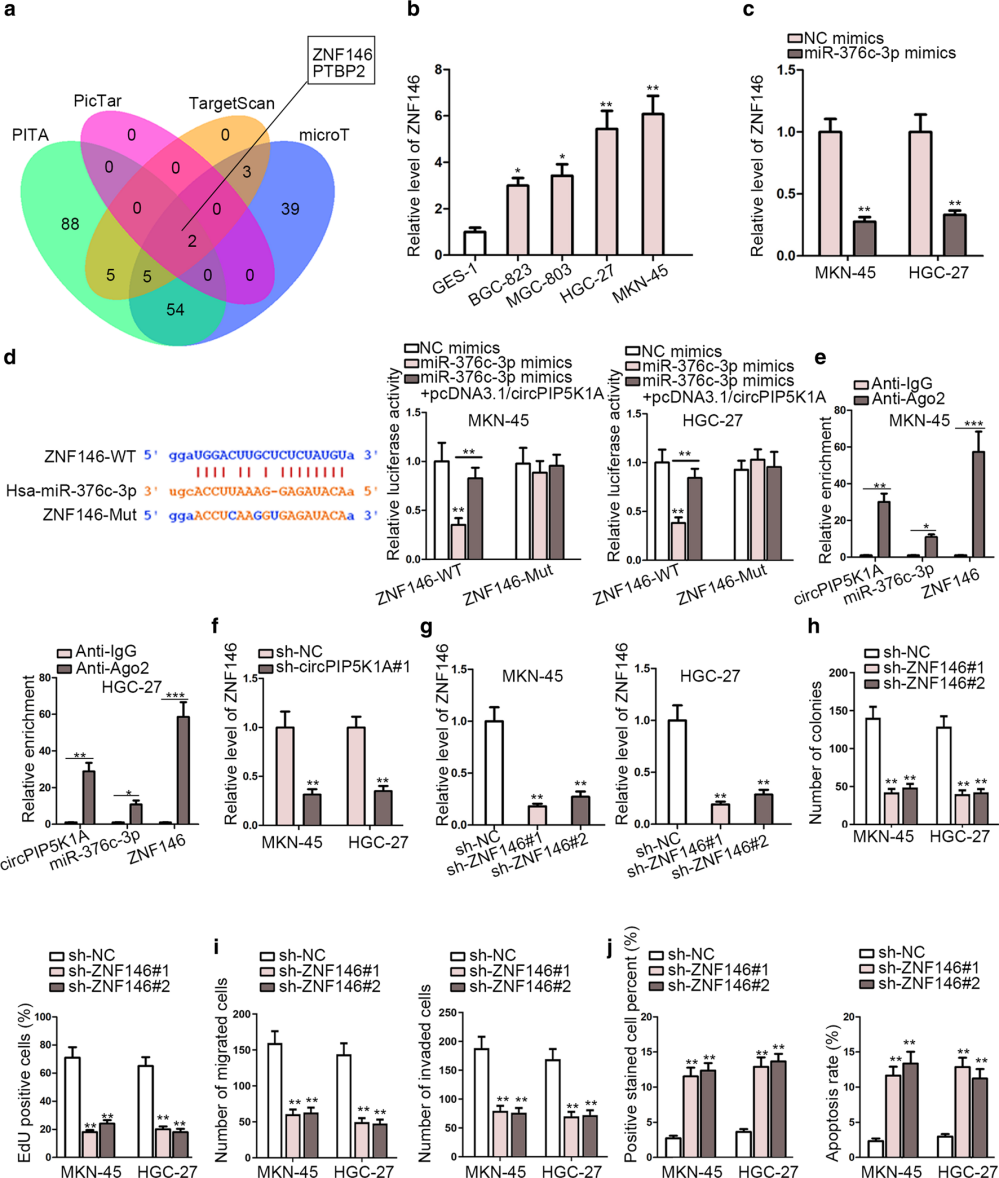
ZNF146 competed with circPIP5K1A for binding with miR-376c-3p. **a** Venn diagram showed the overlapping of four databases (microT, PITA, PicTar and TargetScan), and thus ZNF146 and PTBP2 were discovered to be the candidate target gene of miR-376c-3p. **b** The expression of ZNF146 in GC cell lines and normal GES-1 cells was examined by RT-qPCR. **c** The expression of ZNF146 in transfected cells was detected by RT-qPCR. **d** ZNF146 was predicted to have a binding site for miR-376c-3p from starBase and then luciferase reporter assays were conducted to verify the correlation among circPIP5K1A, miR-376c-3p and ZNF146. **e** RIP assay validated the co-harvest of circPIP5K1A, miR-376c-3p and ZNF146 in RISCs in both MKN-45 and HGC-27 cells. **f** The expression of ZNF146 was detected by RT-qPCR in MKN-45 and HGC-27 cells transfected with sh-circPIP5K1A#1 or sh-NC. **g** The knockdown efficiency of ZNF146 in MKN-45 and HGC-27 cells was detected by RT-qPCR. **h** Colony formation and EdU analyses were applied to measure the proliferative ability of transfected cells. **i** Transwell assay analyzed the capability of cell migration and invasion in transfected cells. **j** TUNEL and flow cytometry analyses were used to examine the apoptosis rate of transfected cells. ^*^P < 0.05, ^**^P < 0.01, ^***^P < 0.001

In depth, we tried to find out the downstream target gene of circPIP5K1A/miR-376c-3p axis in regulating GC cell function. First, Venn diagram showed the overlapping of four databases (microT, PITA, PicTar and TargetScan), and thus ZNF146 and PTBP2 were discovered to be the candidate targets of miR-376c-3p (Fig. 4a). According to GEPIA database, ZNF146 was found to be remarkably upregulated in GC tissues in comparison with adjacent normal tissues whereas PTBP2 expression disclosed no obvious change in theses tissues (Additional file 1: Figure S1). Thus, ZNF146 was undoubtedly selected to be the object of subsequent tests. RT-qPCR detected that the expression of ZNF146 was much higher in GC cell lines than that in normal GES-1 cells (Fig. 4b). Then, an obviously lowered expression of ZNF146 caused by miR-376c-3p upregulation was observed through RT-qPCR analysis in MKN-45 and HGC-27 cells (Fig. 4c). Next, ZNF146 was predicted to have a binding site for miR-376c-3p from starBase and luciferase reporter analysis depicted that circPIP5K1A upregulation counteracted miR-376c-3p upregulation-mediated repressive function on the luciferase activity of pmirGLO-ZNF146-WT (Fig. 4d). Nonetheless, the luciferase activity of pmirGLO-ZNF146-Mut demonstrated no clear change in transfected cells (Fig. 4d). Subsequent RIP assay suggested that circPIP5K1A, miR-376c-3p and ZNF146 were all concentrated in anti-Ago2-precipitated RISCs (RNA-induced silencing complexes) (Fig. 4e). Besides, RT-qPCR obtained a decreased expression of ZNF146 induced by circPIP5K1A deficiency in MKN-45 and HGC-27 cells (Fig. 4f). To make further exploration of the particular role of ZNF146 in GC, functional assays concerning ZNF146 was carried out. Prior to functional tests, RT-qPCR analysis obtained a favorable knockdown efficiency of ZNF146 in MKN-45 and HGC-27 cells (Fig. 4g). Afterwards, colony formation together with EdU analyses observed an evidently weakened ability of cell proliferation imposed by ZNF146 knockdown in MKN-45 and HGC-27 cells (Fig. 4h). Transwell assay uncovered that in MKN-45 and HGC-27 cells ZNF146 knockdown led to an observably attenuated capability of cell migration and invasion (Fig. 4i). Moreover, TUNEL and flow cytometry analysis delineated that down-regulation of ZNF146 accelerated the apoptosis of MKN-45 and HGC-27 cells (Fig. 4j).

### CircPIP5K1A facilitates GC progression by targeting miR-376c-3p/ZNF146 axis

**Fig. 5:**
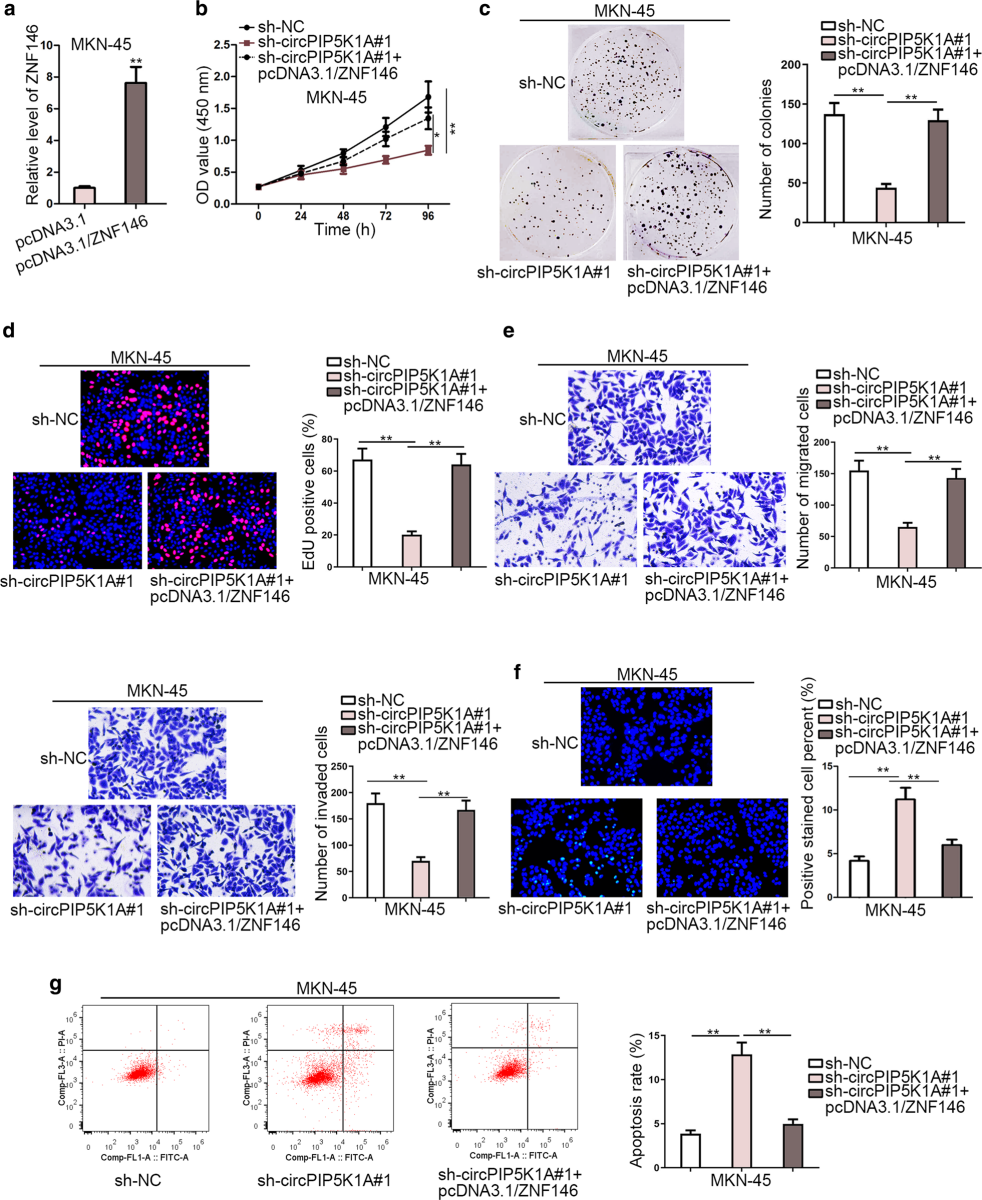
CircPIP5K1A facilitates GC progression by targeting miR-376c-3p/ZNF146 axis. **a** The overexpression efficiency of ZNF146 in MKN-45 cells was detected by RT-qPCR. **b–d** CCK-8, colony formation and EdU analyses were applied to measure the proliferative ability of MKN-45 cells transfected with different plasmids. **e** Transwell assay analyzed the capability of cell migration and invasion in different groups. **f**–**g** TUNEL and flow cytometry assays were used to evaluate the apoptosis rate of transfected cells. ^*^P < 0.05, ^**^P < 0.01

To further prove the ceRNA mechanism of circPIP5K1A in GC, ZNF146 was overexpressed in MKN-45 cells by transfection with pcDNA3.1/ZNF146 at first and then rescue tests were adopted in this study. Figure 5a suggested a satisfactory overexpression efficiency of ZNF146 in MKN-45 cells. As depicted in Fig. 5b–d, cell proliferative ability weakened by circPIP5K1A down-regulation was restored to a great degree after up-regulation of ZNF146. Similarly, the falling trend of cell migration and invasion induced by circPIP5K1A depletion was subsequently recovered by ectopic expression of ZNF146 (Fig. 5e). Last but not least, ZNF146 overexpression largely offset the circPIP5K1A silencing-mediated promoting effect on cell apoptosis (Fig. 5f–g).

### CircPIP5K1A depletion repressed the in vivo tumorigenesis of GC

**Fig. 6:**
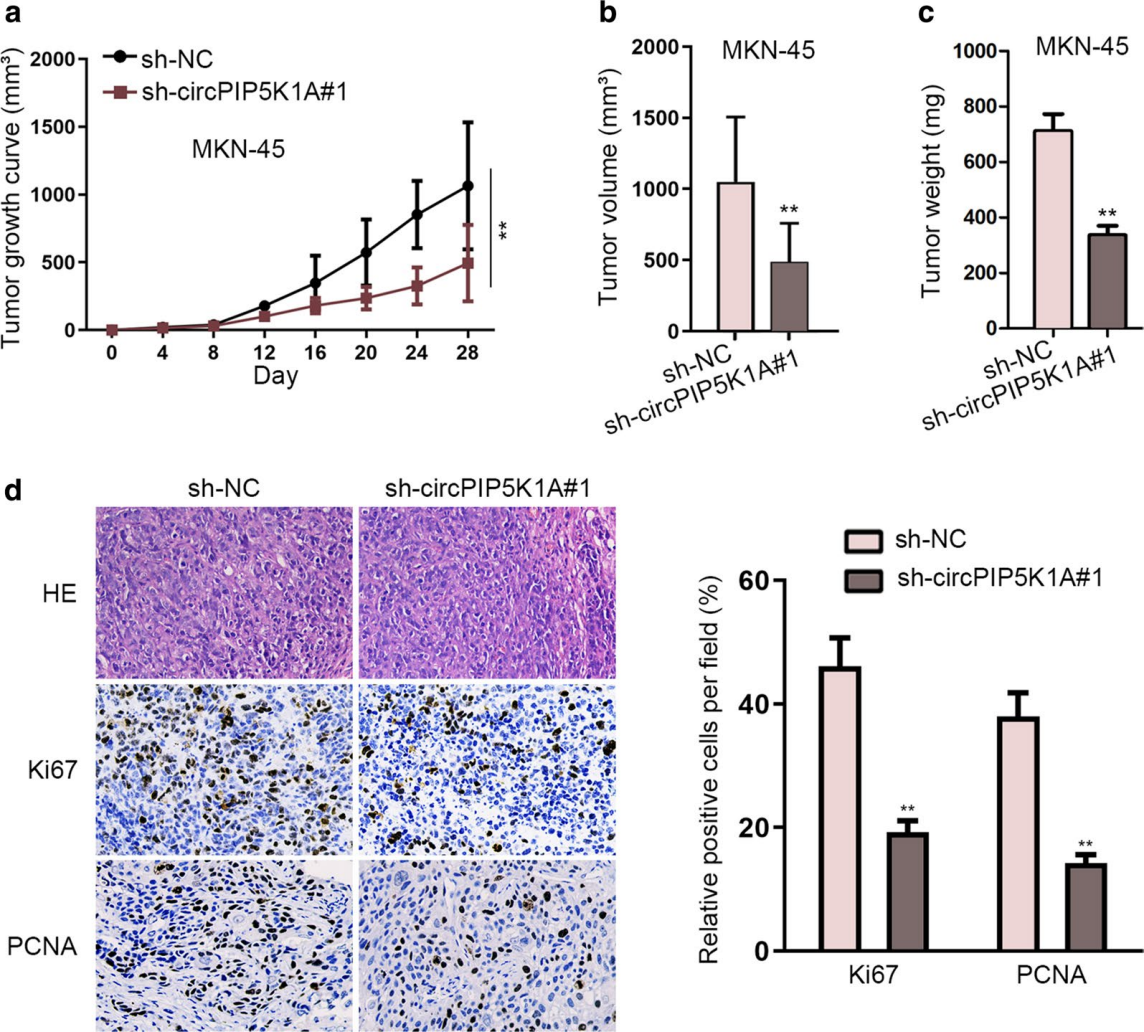
CircPIP5K1A depletion repressed the in vivo tumorigenesis of GC. **a–c** MKN-45 cells transfected with sh-circPIP5K1A#1 or sh-NC were subcutaneously inoculated into the flanks of mice. 28 days later, mice were sacrificed and the in vivo tumorigenesis of GC was studied. Tumor growth, tumor weight and tumor volume in indicated groups were examined. **d** IHC assay was used to evaluate the staining of Ki67 and PCNA in tumors originated from MKN-45 cells with or without circPIP5K1A depletion. ^**^P < 0.01

After experimenting on GC progression in vitro, assays concerning the in vivo tumorigenesis of GC were applied to testify the critical role of circPIP5K1A in GC. MKN-45 cells transfected with sh-circPIP5K1A#1 or sh-NC were subcutaneously inoculated into the flanks of mice. Several days later, mice were sacrificed and the in vivo tumorigenesis of GC was studied. As illustrated in Fig. 6a, knockdown of circPIP5K1A impaired in vivo growth of MKN-45 cells. Additionally, CircPIP5K1A down-regulation led to dramatic decline on the final volume and weight of these in vivo tumors (Fig. 6b–c). Finally, IHC assay suggested that depletion of circPIP5K1A greatly reduced the number of cells with positive Ki67 or PCNA in tumors derived from indicated MKN-45 cells (Fig. 6d).

## Discussion

Diagnosed as a common fatal neoplasm, GC has taken a big proportion in cancer-incurred mortality all over the world [1, 3]. CircRNAs, defined as a new type of endogenous non-coding RNAs with closed-loop structure [13], have been revealed to widely participate in the complex biological courses of diverse tumors, such as breast cancer [17], lung cancer [18], pancreatic cancer [19] and GC [20]. Although a previous research has mentioned the critical role of circPIP5K1A in NSCLC [21], the underlying regulatory mechanism of it in GC remains to be explored. In this study, an obvious high expression of circPIP5K1A in GC tissues and cells was obtained. Additionally, circPIP5K1A was testified to be featured with circular structure in GC. Decreased expression of circPIP5K1A restrained GC cell proliferation, migration and invasion but stimulated cell apoptosis. Taken together, circPIP5K1A elicited oncogenic impact on GC progression.

Mechanically, we speculated that circPIP5K1A might post-transcriptionally modulate gene expression by functioning as a ceRNA in GC based on the data that circPIP5K1A was mainly distributed in cytoplasm, and thereby it was primarily needed to find out the specific miRNA. Subsequently, through bioinformatics prediction and molecular experiments, miR-376c-3p was screened out due to its notable high binding ability with circPIP5K1A and its close association with the progression of human malignancies [25–27]. Then miR-376c-3p was confirmed to be dramatically downregulated in GC cells. And subsequent rescue assays revealed that miR-376c-3p inhibition could largely rescue circPIP5K1A silencing-mediated function on GC progression, which further validated that miR-376c-3p could mediate the function of circPIP5K1A in GC.

Afterwards, four databases (microT, PITA, PicTar and TargetScan) were utilized to find the downstream target gene of miR-376c-3p and thereby ZNF146 and PTBP2 were exhibited through Venn diagram. ZNF146 was later chosen to be studied due to its evident upregulation in GC tissues. Though ZNF146 has been involved in the tumorigenesis of malignant tumors [28, 29], the knowledge of it function in GC remains extremely limited. Thus, tests concerning its expression and potential role in GC were conducted. All the dada from these experiments elucidated that ZNF146 was overtly overexpressed in GC cells and its expression was negatively regulated by miR-376c-3p whereas positively modulated by circPIP5K1A. Besides, ZNF146 depletion impaired GC tumorigenesis and development. Moreover, rescue assays depicted that ZNF146 overexpression could largely offset the circPIP5K1A silencing-mediated effect on GC progression. After experiments concerning the progression of GC in vitro, assays related to the in vivo tumorigenesis of GC were applied and thus further proved the oncogenic role of circPIP5K1A in GC.

## Conclusion

Combining all these findings, a conclusion can be reached that circPIP5K1A facilitates GC progression via miR-376c-3p/ZNF146 axis, illuminating that circPIP5K1A might be capable of functioning as a new therapeutic target for GC treatment.

## Supplementary information

**Additional file 1: Figure S1**. The expression of ZNF146 and PTBP2 in 408 GC tissues relative to 211 normal gastric tissues was obtained from GEPIA database.

## Data Availability

Research data and material are not shared.

## Abbreviations

circRNAs: Circular RNAs
GC: Gastric cancer
NSCLC: Non-small cell lung cancer
ActD: Actinomycin D
RISC: RNA-induced silencing complex
ceRNA: Competing endogenous RNA
