# Supplementary figures and images for: CircPIP5K1A facilitates gastric cancer progression via miR-376c-3p/ZNF146 axis
Source: Cancer Cell Int. 2020 Mar 14;20:81. doi: 10.1186/s12935-020-1122-5 (PMC7071687; doi:10.1186/s12935-020-1122-5)

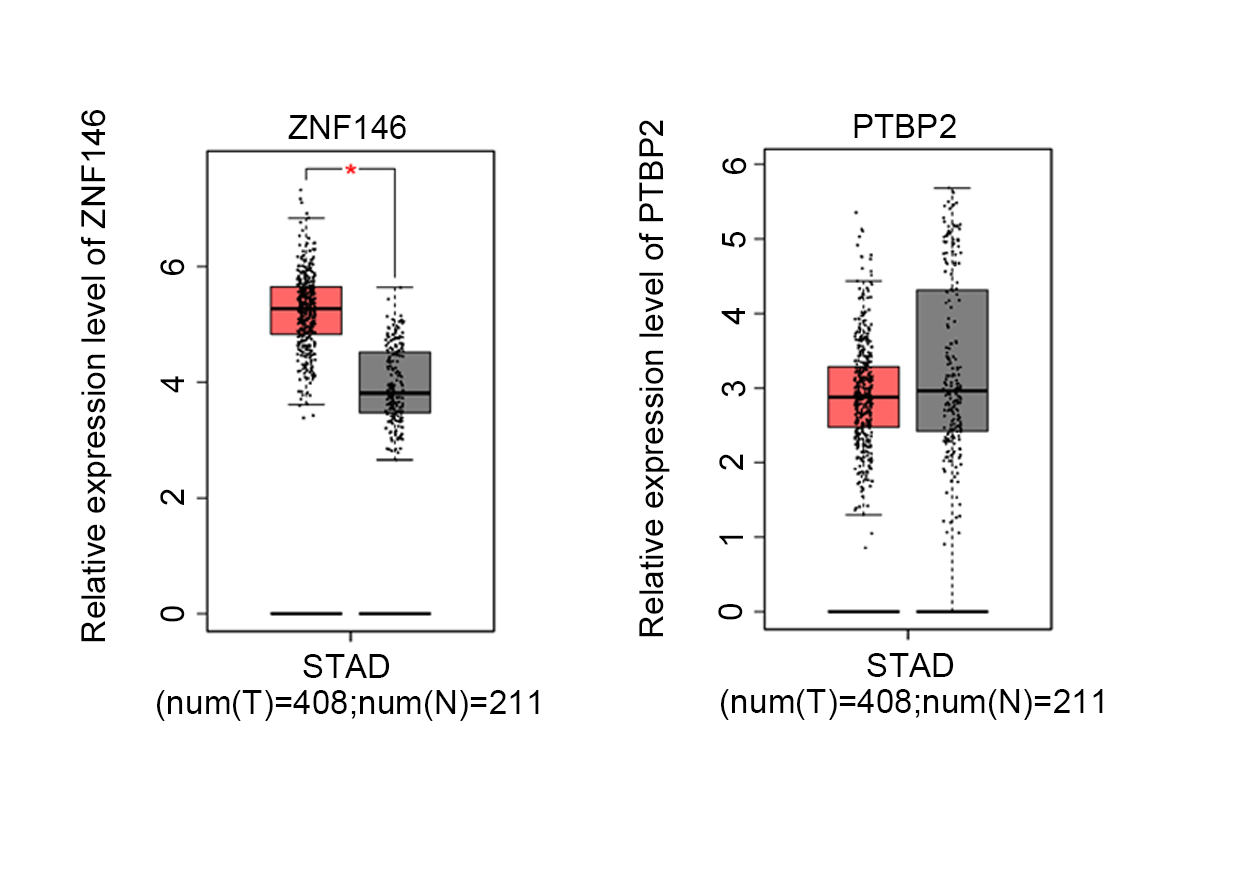

Supplement: Supplementary file 1 — Additional file 1: Figure S1. The expression of ZNF146 and PTBP2 in 408 GC tissues relative to 211 normal gastric tissues was obtained from GEPIA database. [file 12935_2020_1122_MOESM1_ESM.tif]
